# Metabolomics Identifies Biomarker Pattern for Early Diagnosis of Hepatocellular Carcinoma: from Diethylnitrosamine Treated Rats to Patients

**DOI:** 10.1038/srep16101

**Published:** 2015-11-03

**Authors:** Jun Zeng, Xin Huang, Lina Zhou, Yexiong Tan, Chunxiu Hu, Xiaomei Wang, Junqi Niu, Hongyang Wang, Xiaohui Lin, Peiyuan Yin

**Affiliations:** 1 Key Laboratory of Separation Science for Analytical Chemistry, Dalian Institute of Chemical Physics, Chinese Academy of Sciences, Dalian 116023, China; 2 School of Computer Science & Technology, Dalian University of Technology, 116024 Dalian, China; 3 International Cooperation Laboratory on Signal Transduction, Eastern Hepatobiliary Surgery Institute, the Second Military Medical University, Shanghai, China; 4 Department of Hepatology, First Hospital, Jilin University, Changchun, Jilin 130021, China

## Abstract

Early diagnosis of hepatocellular carcinoma (HCC) remains challenging to date. Characteristic metabolic deregulations of HCC may enable novel biomarkers discovery for early diagnosis. A capillary electrophoresis-time of flight mass spectrometry (CE-TOF/MS)-based metabolomics approach was performed to discover and validate potential biomarkers for HCC from the diethylnitrosamine-induced rat hepatocarcinogenesis model to human subjects. Time series sera from the animal model were evaluated using multivariate and univariate analyses to reveal dynamic metabolic changes. Two independent human cohorts (populations I and II) containing 122 human serum specimens were enrolled for validations. A novel biomarker pattern of ratio creatine/betaine which reflects the balance of methylation was identified. This biomarker pattern achieved effective classification of pre-HCC and HCC stages in animal model. It was still effective in the diagnosis of HCC from high-risk patients with cirrhotic nodules, achieving AUC values of 0.865 and 0.905 for two validation cohorts, respectively. The diagnosis of small HCC from cirrhosis with an AUC of 0.928 highlighted the potential for early diagnosis. This ratio biomarker can also improve the diagnostic performance of α-fetoprotein (AFP). This study demonstrates the efficacy of present strategy for biomarker discovery, and the potential of metabolomics approach to provide novel insights for disease study.

Hepatocellular carcinoma (HCC) is the most common liver neoplasm with high lethality (<7% of a five-year survival rate)^1^. Most of the burden of HCC is in developing countries, especially in East Asia and Africa with high incidence (>20 per 100,000 individuals)^2^,^3^. The majority of HCC cases develop from precancerous lesion of liver cirrhosis^4^,^5^. Thus screening of HCC at early stage is an effective strategy to decrease the high mortality. Due to the unapparent early symptoms of HCC, regular imageology (i.e., ultrasonography) and serology (α-fetoprotein, AFP) inspection are the major screening methods for HCC. The screening of HCC has improved survival rates of patients in the past thirty years, however, early and accurate diagnosis of HCC is still a great challenge until now^6^,^7^. Novel screening methods are still intensively needed especially for the discrimination of patients with cirrhotic nodules and small malignant HCC^8^.

Apart from the changes of gene and protein, reprogrammed metabolism is one of important characteristics of tumor cell^9^. Cancer cells reprogram their metabolism to meet the requirements of malignant proliferation and metastasis. The reprogrammed metabolism can then be analyzed to understand the process of carcinogenesis, and provide valuable clue to discovering potential metabolic biomarkers for early diagnosis. Metabolomics has been increasingly employed as an attractive platform to monitor and screen the reprogrammed metabolic profiling of tumor^10^,^11^,^12^.

To date, a series of studies about individual metabolites or their combination, such as serum γ-glutamyl dipeptides^13^, bile acids^2^, fatty acids^14^, sphingosines^15^ and urine carnitine^16^, have been reported to distinguish different forms of hepatic disease. These studies enrich the pool of potential biomarkers for future clinical application. It is worth noting that most of these potential biomarkers were found from typical populations such as HCC and non-HCC controls, reflecting the typical metabolic phenotypes of HCC cohort after the occurrence of tumors. However, few dynamic metabolic changes which indicate the process of carcinogenesis were evaluated. Thus early biomarkers with the tendency of tumorigenesis cannot be easily found from dozens of differential metabolites. Time series samples would be necessary for such dynamic metabolomics study, which would be possible to provide insight into the interfacial stage between precancerous cirrhosis and HCC, and further facilitate the screening of biomarkers for early diagnosis. However, it would be a hard work to collect sequencing samples from high-risk populations. Due to the similarity to histological and genetic features of patients^17^, animal models, such as diethylnitrosamine (DEN)-induced HCC model, are commonly used to imitate the process of stepwise hepatocarcinogenesis from cirrhosis^5^,^18^,^19^.

In our previous report^20^, the DEN-induced rat hepatocarcinogenesis model was employed and analyzed by using liquid chromatography-mass spectrometry (LC-MS) to obtain time-related hydrophobic metabolic features and potential biomarkers. Our findings indicated that the metabolic deregulations in rat models are similar to patients, and it would be possible to discover potential biomarkers for early diagnosis. However, due to the limitation of the analysis method, polar metabolic profiling was seldom depicted in previous work about DEN-induced hepatocarcinogenesis model^20^. In recent years, since polar and ionic metabolites are well recognized for tumorigenesis, suppression and signaling^10^,^11^,^21^,^22^, more such metabolites should be concerned in subsequent studies.

Therefore, in this study, the DEN-induced HCC model was developed and analyzed using capillary electrophoresis-time of flight mass spectrometry (CE-TOF/MS)-based polar metabolomics approach. This present study aims to depict the the process of hepatocarcinogenesis, and extend the scope of potential biomarkers from novel perspective of polar metabolomics. Time series serum samples from DEN animal model were analyzed to evaluate the dynamic metabolic changes and discover biomarker candidates. Considering the differences between rats and human, and between chemical induction and viral infection, the potential biomarkers were further validated in two independent human cohorts. The efficacy of potential biomarkers for early diagnosis was evaluated by distinguishing high-risk populations of cirrhosis from HCC, especially small HCC.

## Results

**Figure 1:**
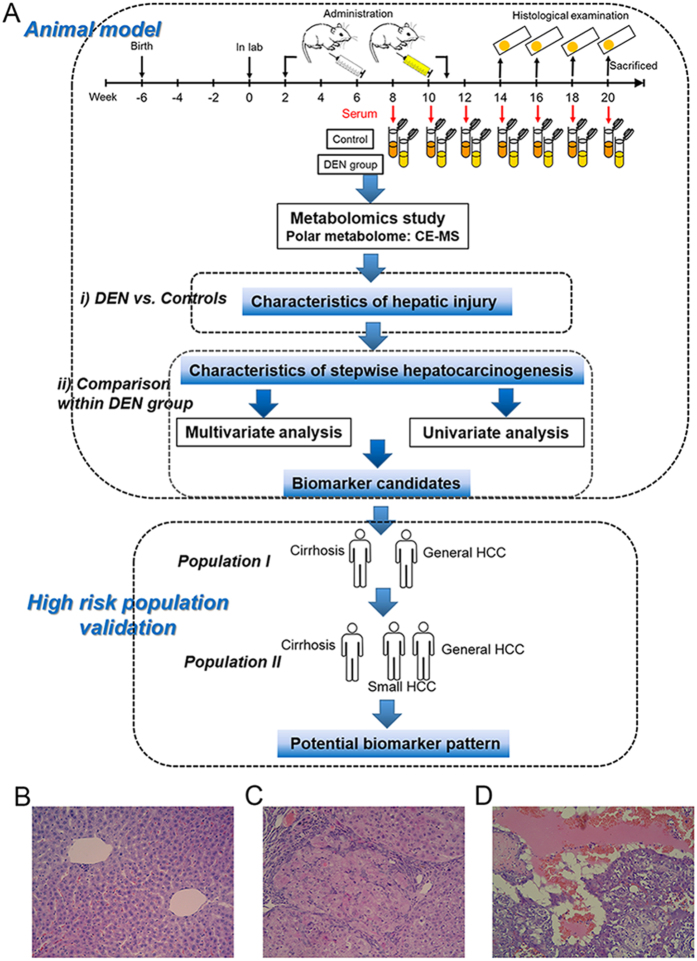
Study scheme and animal modeling. (**A**) Scheme of metabolomics study. (**B-D**) are typical microphotographs of normal, cirrhotic and HCC liver tissue (hematoxylin and eosin (H&E), original magnification ×20). Images drawn by Jun Zeng.

The scheme of metabolomics study on biomarker discovery and validation is given in Fig. 1A.

### Histological characteristics of animal model

As shown in Table S1, the comparison between model and control rats both at week 20 reveals that there were significant DEN-induced decrease in body weight and increase in relative weight of liver (*p* < 0.05). No significant difference was found in DEN group from week 14 to week 20 in the relative weight of liver.

Based on the histological results of our previous experiment (weeks 2–20)^20^, the liver carcinogenesis was found from week 14 to week 20 during modeling. In the modeling of this present study, histological examinations indicated that sacrificed DEN rats at week 14 exhibited characteristic histological changes of cirrhosis, except that only one rat was observed in developing liver tumor. Sacrificed DEN rats exhibited progressive incidences of the liver tumor from week 16 to week 18. And all 7 DEN rats alive at the end of the study (week 20) were tumor-bearing. Typical histological microphotographs of normal liver, cirrhotic liver and tumor are shown in Fig. 1B–D. Histological sections verified that the DEN-induced hepatocarcinogenesis model was successfully produced in this study.

### The metabolic deregulations of rat model

Time-series sera from model and control animals were collected for metabolomics study. There were 184 polar metabolites measured from rat sera based on our metabolic profiling strategy of peak identification and refining^21^, including amino acids, polypeptides, amines, nucleosides, carbohydrates, organic acids and etc. The quality of acquired metabolic profiling was monitored by evaluating quality control samples (QCs) and confirmed to be satisfactory based on our published methods^21^ (Fig. S1).

**Figure 2:**
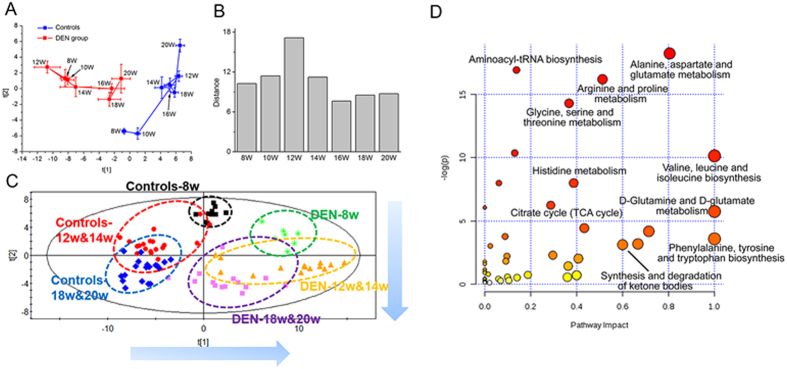
Metabolic profiling and comparison between model and control rats. (**A**) Metabolic trajectories of control and DEN rats based on PCA. Each point represents the average score values of samples with standard error (SE). (**B**) Euclidean distance between control and DEN groups. (**C**) Metabolic changes of the animal model. This PLS-DA model passed cross validation without overfitting. (**D**) The most relevantly disturbed pathways.

Considering the histological results of the present and our previous modeling^20^, the serial serum set was divided into three stages: week 8 (the inflammation stage), weeks 10–14 (the cirrhosis stage) and weeks 16–20 (the HCC stage). The metabolic trajectories of DEN and control rats were depicted from weeks 8 to 20 based on PCA model (Fig. 2A). To determine the metabolic differences of these two trajectories (models vs. controls), the Euclidean distance between the control and DEN groups at each time point was further calculated (Fig. 2B). The difference was most evident at week 12, one week after the last administration of DEN at week 11, indicating severe metabolic deregulations of model rats. The magnitude of metabolic changes in the HCC stage (weeks 16–20) was lower than those in the pre-HCC stage (weeks 8–14). Furthermore, a PLS-DA model was used to evaluate the characteristic changes among different stages without considering samples from interfacial stages (week 10 and week 16). Two types of metabolic disturbance, including DEN-induced changes and age-related changes, could be observed on the score plot along the first and second principal component directions, respectively (Fig. 2C).

Prior to univariate statistical test, metabolites with large measurement error (RSD of area in QC samples >30%) were removed from the data set. A total of 115 significant differential metabolites (Wilcoxon Mann-Whitney test, *p* < 0.05) were refined from the comparisons between model and control groups at all 7 time points (i.e., the union of 7 comparisons, Table S5), which revealed that alanine, aspartate and glutamate metabolism, arginine, serine and threonine metabolism, valine, leucine and isoleucine biosynthesis, TCA cycle and so on were most relevantly disturbed due to the DEN-induced hepatocellular damage (Fig. 2D).

To further subtract the metabolic disturbance of model rats from the baseline level of controls (i.e., remove age-related changes), the contents of metabolites for each DEN sample were divided by the average of age-matched controls. The new conversion dataset with the relative contents of 115 screened significant differential metabolites was used for subsequent statistical study (Table S5).

### Dynamic metabolomics changes associated with hepatocarcinogenesis

Dynamic metabolomics changes associated with hepatocarcinogenesis were analyzed based on the comparisons within DEN samples across seven time points.

**Figure 3:**
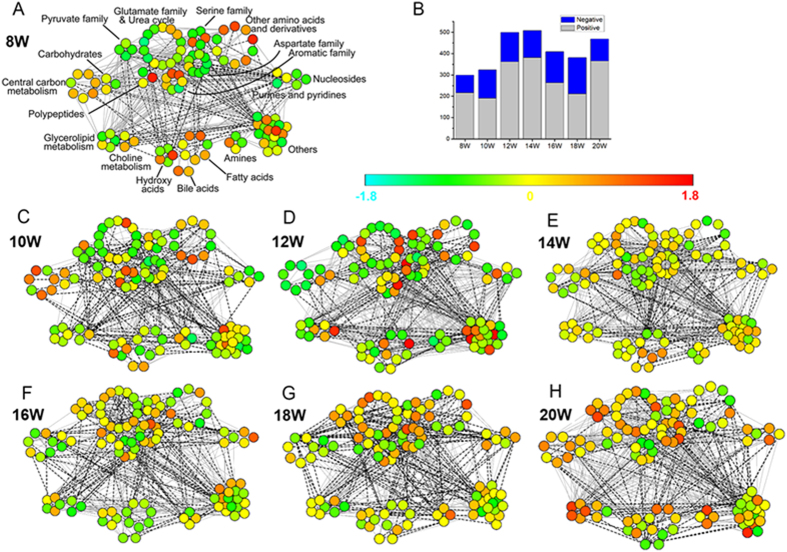
Correlation network analysis of carcinogenesis stages. (**A**,**C**–**H**) are correlation networks (weeks 8–20). Each point represents one metabolite with the relative content of UV scaled. Each grey solid (or black dotted) line represents the positive (or negative) correlation with |Cij| >0.8. (**B**) The number of lines at each time point.

Correlation network analysis was performed to reveal the progressive profiles of different classes of metabolites. At each time point, the Pearson correlation coefficient between metabolites was calculated using relative contents of differential metabolites (Table S5). As shown in Fig. 3, each point represents one metabolite with unit variance (UV) scaling, and the line represents the correlation with |Cij| > 0.8. The number of lines evidently elevated during week 12 to 14 (cirrhosis stage), and subsequently mitigated from week 16 (early HCC stage), until week 20 (final HCC stage) with a re-increase (Fig. 3B). The results of lines reveal the increased correlation between metabolites from week 12, confirming the activated metabolic disturbance in the cirrhosis stage. Furthermore, from the perspective of “point”, it can be observed that different classes of metabolites exhibited different changing characteristics. Metabolites from central carbon metabolism showed a sharp decrease at week 12 (middle cirrhosis stage), and then elevated. Most of amino acids (such as pyruvate family, serine family, aspartate family and glutamate family), amines and metabolites from glycerolipid metabolism were subjected to increase from week 14 (final cirrhosis stage), and obtain the highest levels at week 20 (final HCC stage). Nucleosides, purines and pyridines exhibited higher levels in the HCC stage. These changes indicate the progression of DEN-induced hepatocarcinogenesis.

Subsequently, two PLS-DA models were built using the conversion dataset to distinguish stages within DEN samples (Fig. S2A,B). Without considering samples from interfacial stages, the model I was used for the classification of inflammation (week 8), cirrhosis (weeks 12 to 14) and HCC (weeks 18 to 20) samples, while the model II was used for the categorization of pre-HCC (weeks 8–14) and HCC (weeks 18–20) samples. A clear separation could be observed among the three stages of inflammation, cirrhosis and HCC. More importantly, the separation between pre-HCC and HCC stages is also obviously represented. Then, a total of 76 metabolites were further refined with variable importance in the projection (VIP) value exceeding 1 for either PLS-DA model. These metabolites had the above statistical importance on the classification with a better reflection of metabolic trends regarding tumorigenesis.

**Figure 4:**
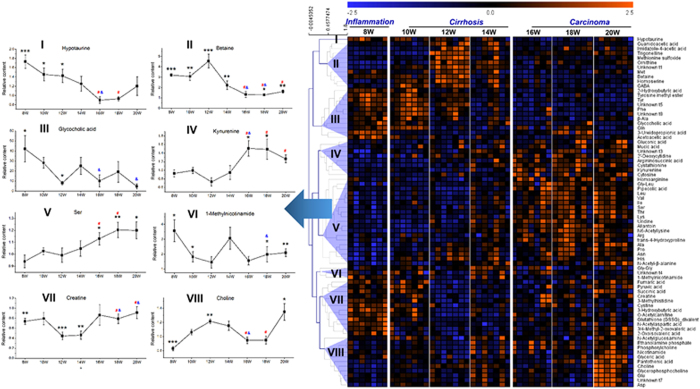
Metabolites clustering and response trajectory analysis. Defined metabolites were clustered based on the similarity of response pattern (right panel). Representative metabolites were selected from each cluster to present the response trajectory (left panel). Each point in the trajectory was presented as the average relative content ± SE. The black * means the statistical significance between DEN group and age-matched controls. The red # (or blue &) means the statistical significance between week 12 (or week 14) and the corresponding week. *^,#,&^0.01 < p < 0.05, **0.001 < p < 0.01 and ***p < 0.001.

These differential metabolites were subjected to HCA to further specialize the response trajectory across different stages. These metabolites were clustered into eight major groups according to the similarity of response pattern (Fig. 4). The representative metabolites were selected from each cluster to present the trajectories associated with hepatocarcinogenesis. Besides the graded response trajectories, nonmonotonic response curves also existed due to the complexity of hepatocarcinogenesis.

### Screening of biomarkers candidates from DEN animal model

**Figure 5:**
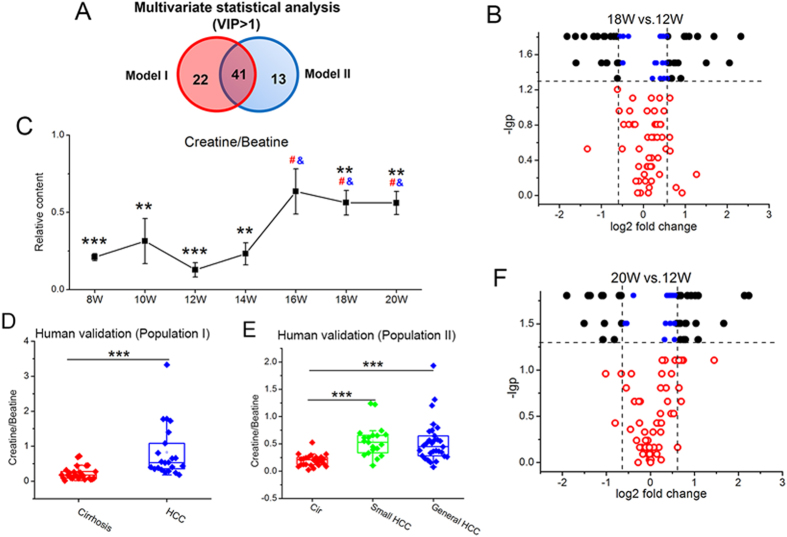
Screening and validation of potential biomarkers. (**A**) Venn diagram. (**B**,**F**) are volcano plots for the comparisons of significant differential metabolites (Table S5). (**C**) The response trajectory of the biomarker pattern (i.e., ratio creatine/betaine). (**D**,**E**) are box plots of this ratio biomarker for population I and II.

As shown in the Venn diagram (Fig. 5A), 76 metabolites associated with hepatocarcinogenesis were refined from the DEN-induced significant metabolic changes (Table S5) based on the VIP values of PLS-DA models. To narrow down the scope of biomarker candidates, the intersection of 41 metabolites with VIP > 1 in both two models was finally retained from the multivariate screening.

Then, univariate statistical analysis was performed to confirm the metabolic differences between HCC and pre-HCC stages. Considering the most evident metabolic disturbance at week 12 (the middle cirrhosis stage), metabolic profiling was firstly compared between typical samples from week 12 and the other two HCC cohorts from week 18 and week 20, respectively. 34 metabolites significantly changed in both comparisons with −lg*p* > 1.3 (i.e., Wilcoxon Matched-Pairs Signed-Ranks test, *p* < 0.05, Fig. 5B,F). These metabolites were considered to be of representative differential characteristics between HCC and pre-HCC cirrhosis. The fold change of these 34 metabolites was subsequently taken into consideration. Based on the volcano plots (Fig. 5B,F), 18 metabolites were further retained with |log2 fold change|>0.585 (i.e., fold change > 3/2 or fold change < 2/3).

Taken together, 8 metabolites were cross-selected based on the preliminary screening of multivariate and univariate analysis.

To examine the potential of these 8 metabolites for early HCC discrimination, especially distinguishing borderline stages, the evaluation of statistical importance was moved forward to the comparison between week 14 (the final cirrhosis phase) and the other two early HCC cohorts of week 16 and week 18, respectively. Finally, 5 metabolites including betaine, creatine, kynurenine, pipecolic acid and one unidentified metabolites (unknown 1) were confirmed with statistical significance (*p* < 0.05) for either comparison. They were regarded as biomarker candidates from the DEN animal model.

### Preclinical validation and evaluation of potential biomarker

External validations for high-risk populations of cirrhosis and HCC patients were performed to test the efficacy of 5 candidates discovered from animal experiment.

The result of population I (25 patients with cirrhosis and 22 patients with HCC) indicated the usefulness of betaine and creatine (cirrhosis vs. HCC, *p* < 0.05). Another population set (population II) consisting of 50 HCC patients (20 small HCC and 30 general HCC patients) and 25 cirrhosis patients in another batch was analyzed for further validation. The result of population II re-confirmed the statistical significance (*p* < 0.05) of these two metabolites when comparing cirrhosis with HCC, especially small HCC subjects.

It was observed that betaine and creatine came from different clusters with opposite change trends during the process of hepatocarcinogenesis from cirrhosis to HCC (Fig. 4). In addition, these two metabolites were closely related in the pathway^23^. Therefore, the ratio of these two metabolites was calculated to develop a new biomarker pattern (i.e., ratio creatine/betaine). The statistical results of this new potential biomarker pattern were presented in Fig. 5. In the animal model, this ratio biomarker with relative content (i.e., normalization using the average of age-matched controls) can be observed with significant difference (p < 0.05) between cirrhosis and HCC from the early HCC phase (week 16, Fig. 5C). Similar change trend and statistical significance were also obtained from two independent human populations (*p* = 1.82E-05 for cirrhosis vs. HCC in population I and *p* = 2.21E-06 for cirrhosis vs. general HCC in population II, Fig. 5D,E). Notably, it is still effective for the discrimination of small HCC (*p* = 2.27E-06 in population II, Fig. 5E).

**Figure 6:**
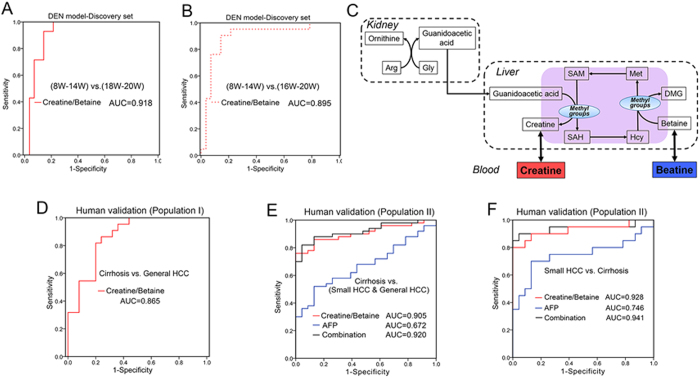
Evaluation of potential biomarker. (**A**,**B**,**D**–**F**) are ROC curves. Diagnostic potential was evaluated based on binary logistic regression. (**C**) Metabolism of betaine and creatine. Arg, arginine; Gly, glycine; Met, methionine; SAM, S-adenosylmethionine; SAH, S-adenosylhomocysteine; Hcy, homocysteine; DMG, dimethylglycine.

The diagnostic potential of this biomarker pattern was then carefully evaluated based on the ROC curve with the AUC (area under the curve) value, the sensitivity and specificity at best cut-off points (Table 1, Fig. 6A,B,D–F). In the animal model, this ratio biomarker achieved an AUC value of 0.918 in the discrimination of weeks 8–14 (pre-HCC stage) and weeks 18–20 (HCC stage), and the sensitivity and specificity were 92.9% and 85.7%, respectively (Fig. 6A). When the borderline stage of week 16 (early HCC phase) was taken into consideration, 90.5% of samples from weeks 16–20 and 85.7% of samples from weeks 8–14 were correctly discriminated at the best cutoff point, with an AUC value of 0.895 (Fig. 6B). Next, the evaluation was extended to the patients with HCC and the high-risk populations of cirrhosis. This ratio biomarker was effective in the diagnosis of human subjects with an AUC value of 0.865 and 0.905 for populations I and II, respectively (Fig. 6D,E). The potential of early diagnosis was further highlighted for a more difficult diagnosis of small HCC from pre-cancer cirrhosis (population II), achieving an AUC value of 0.928 (Fig. 6F).

The diagnostic performance of this metabolic biomarker was compared with traditional AFP. When complete HCC samples (including small HCC and general HCC, population II) were subjected to the comparison, we found that the AUC result of this biomarker pattern (AUC = 0.905) was better than that of AFP (AUC = 0.672) (Fig. 6E). Similar comparison result for the diagnosis of small HCC and cirrhosis was also obtained (Fig. 6F). The combination of this metabolic biomarker and AFP achieved new AUC values of 0.920 for all HCC (Fig. 6E) and 0.941 for small HCC (Fig. 6F), which greatly improved the diagnostic performance of traditional AFP. The combinational use of them has the promising clinical potential to improve the diagnostic accuracy of HCC.

## Discussion

Until now, early diagnosis of HCC is still a great challenge. Considering the fact that it is difficult to collect samples from HCC patients at a very early stage due to the fast development of tumorigenesis and challenging early discovery, the DEN animal model was employed to imitate the process of stepwise hepatocarcinogenesis. The strategy was demonstrated to be practical in our former study^20^. Here, the histological examination confirmed that the DEN-induced hepatocarcinogenesis model was successfully established. Then, time series sera from this animal model were analyzed to reveal the dynamic changes of polar metabolites during the hepatocarcinogenesis. Refined potential biomarkers were further validated in two human cohorts. The efficacy for early diagnosis was evaluated by distinguishing high-risk populations of cirrhosis from HCC, especially small HCC. This dynamic discovery strategy based on time series evaluation provided an opportunity to recognize the metabolic changes across tumorigenesis process, especially to focus on the interfacial stage between precancerous cirrhosis and HCC, which greatly facilitated the screening of potential biomarkers for early diagnosis.

Exposure to DEN has been reported to accumulate extra reactive oxygen species (ROS) in hepatic cells^24^,^25^. The balance between ROS production and cell antioxidant capability was disturbed, resulting in oxidative stress and DNA damage, ultimately triggering liver carcinogenesis^25^,^26^. The oxidative stress may inflict damages to the metabolism of hepatic cells. Pathway analysis has revealed that due to the DEN-induced hepatocellular damage, several metabolic pathways including alanine, aspartate and glutamate metabolism, arginine, serine and threonine metabolism, valine, leucine and isoleucine biosynthesis, TCA cycle and so on were most relevantly disturbed (Fig. 2D). It can be observed that different classes of metabolites exhibited various patterns with the progression of hepatocarcinogenesis. Obviously, most amino acids (such as pyruvate family, serine family, aspartate family and glutamate family), amines and metabolites from glycerolipid metabolism were subjected to increase from the final cirrhosis stage (week 14), and obtain the highest levels at the final HCC stage (week 20). From the perspective of variation amplitude, the summaries of trajectories for samples (Fig. 2B) and metabolites (Fig. 4B) suggested the more vigorous metabolic disturbance for the pre-cancer cirrhosis stage. The relatively low metabolic disturbance for cancer stage may attribute to the reduction of antioxidant activity in DEN-exposed groups^25^. With the increase of abnormal cell (i.e. preneoplastic and neoplastic cell), the expression of antioxidant gene may deregulate, decreasing the antioxidant reactions of liver to defense to excessive ROS production^25^.

Although the metabolic profiles could clearly separate HCC and cirrhosis, it is still not feasible to use the complex metabolomics data matrix in the clinic. Thus we tried to refine biomarker patterns out of hundreds of differential metabolites. The discovery of biomarker candidates from serial sera consisted of a two-step comparison: i) background subtraction. To revel characteristics associated with DEN-induced hepatic injury, 115 differential metabolites with statistical significance (*p* < 0.05) were firstly acquired based on the comparison between model and age-matched control rats. To reduce the influence of age for stepwise metabolic changes, the contents of metabolites from each DEN sample were then normalized using the average of controls at the same age (i.e., the relative content). ii) Metabolic characteristics filtration. These differential metabolites were further explored based on the comparison within DEN samples across seven time points, in order to pick up characteristics associated with stepwise hepatocarcinogenesis. A comprehensive workflow was employed to determinate potential biomarkers, including the visualization of trajectories for samples (Fig. 2A) and metabolites (Fig. 4), multivariate screening for the classification of different disease status, stepwise univariate judgment for the discrimination of important stages and external validation in high-risk human cohorts. Two metabolites (betaine and creatine) were defined as biomarker candidates.

Metabolic pathway analysis indicated that betaine plays an essential role in the maintenance of hepatic methyl balance^27^. As an important methyl donor, betaine participates in the formation of methionine, subsequently S-Adenosylmethionine (SAM). SAM donates the methyl group to substrates via numerous methyltransferases, and one of the important transmethylation reactions is the methylation of guanidoacetic acid to form creatine^28^ (Fig. 6C). Betaine and creatine are representative methyl donor and acceptor, respectively. During the process of hepatocarcinogenesis from cirrhosis to HCC, these two metabolites came from different clusters (Fig. 4) of trajectories with opposite change trends. The significant change of ratio creatine/betaine across the hepatocarcinogenesis process may indicate the disruption of hepatic methyl balance, demonstrating the biological implication of this potential biomarker pattern. As it is reported, the methyl balance is of great significance in supporting normal liver function, and the imbalance is associated with abnormal DNA synthesis, aberrant methylation reactions, or oxidative stress^23^. Therefore, the ratio of creatine/betaine was combined as candidate biomarker pattern for the early diagnosis of HCC. This potential biomarker pattern may has the advantages of amplifying the metabolic difference for discrimination, improving the diagnostic performance, simplifying the practical application and indicating the change of related metabolic pathway.

The diagnostic potential of this biomarker pattern was also evaluated. In animal model, this biomarker pattern can achieve effective classification of pre-HCC and HCC stages (Fig. 6A,B). Subsequently, the evaluation was extended to the human subjects. This ratio biomarker was still effective in the diagnosis of high-risk subjects with cirrhotic nodules and malignant HCC (Fig. 6D,E). Especially, the diagnosis of small HCC from pre-cancer cirrhosis with an AUC value of 0.928 indicated the potential for early diagnosis (Fig. 6F). Moreover, this biomarker pattern greatly improved the diagnostic performance of traditional AFP. The combinational use of them has the promising clinical potential to improve the diagnostic accuracy of HCC. More studies are still needed for further large-scale validation.

In summary, the discovery of biomarkers for early diagnosis started with the study of the DEN-induced stepwise hepatocarcinogenesis model, and then extended to the validation in typical human populations. Small HCC subjects were also particularly enrolled to evaluate the potential of early diagnosis. Compared with previous reports of typical populations^2^,^13^, this dynamic metabolomics study of the stepwise hepatocarcinogenesis process would prefer to the screening of early metabolic features for HCC. Besides, this CE-TOF/MS-based metabolomics study supplements the knowledge about hepatocarcinogenesis, and extend the scope of potential biomarkers from the novel perspective of polar metabolomics. This study identified a novel ratio biomarker pattern of creatine/betaine, which related with the disruption of hepatic methyl balance of HCC individuals. This ratio biomarker achieves effective classification of the disease stage in animal model, and was also evaluated as a good tool in diagnosing patients with pre-cancer cirrhosis and HCC, especially small HCC. Furthermore, this ratio biomarker can greatly improve the diagnostic performance of traditional AFP, indicating the potential of the combinational use of them to improve the diagnostic accuracy of HCC. The presented strategy for biomarker discovery refined and simplified the usage of complex metabolomics data. And the measurement of the ratio of two metabolites would be practical for validations and future clinical applications.

## Methods

### Animals and DEN treatment

The animal experiment was conducted at Dalian Medical University (Dalian, China), complying with the national guidelines for the care and use of laboratory animal. All animal experiment were approved the experimental animal ethics committee of Dalian Medical University. A total of 55 male Sprague-Dawley rats were enrolled at the age of 42 days. Scheme of the animal experiment is given in Fig. 1A.

Rat hepatocarcinogenesis model was established as our previous report^20^. Briefly, week 0 was defined as the starting time point of animal experiment. Then, after two weeks of adaptive inhabitation, all rats were randomly divided into two groups: control group (n = 10) and model group (n = 45). From week 2 to week 11, model rats were administrated with DEN (70 mg/kg body weight) via intraperitoneal injection once a week, while control rats received saline injections of equivalent volumes. 14 rats from the model group died during the administration. Standard commercial laboratory rat chow was used and water was freely available in the animal experiment.

To monitor the progress of stepwise hepatocarcinogenesis, 8 rats from the model group were sacrificed for histological examination every 2 weeks from week 14 to week 20. In week 20, all surviving rats (n = 7 for model group and n = 10 for control group) were finally sacrificed. Collected liver tissues were weighted (Table S1), and then fixed in 10% buffered formalin for histopathology examination. A time-serial sera set, including 7 rats from model group and 10 rats from control group, was collected once every 2 weeks from week 8 to week 20. All 119 rat serum specimens from 7 time points were stored immediately at −80 °C.

### Human specimens

A total of 122 human serum specimens were enrolled under fasting condition with written informed consent. The study protocol was reviewed and approved by the institutional reviewer board of Eastern Hepatobiliary Surgery Institute, the Second Military Medical University, Shanghai, China. The experiment was carried out in accordance with the approved guidelines.

The first population set (population I) includes 22 patients with HCC and 25 patients with cirrhosis. Another 50 HCC patients and 25 cirrhosis patients consist of the second population set (population II). All HCC samples were collected from the National Liver Tissue Bank in the Second Military Medical University (Shanghai, China) with histopathologically diagnosed after tumor excision. The second validation population contains 20 small HCC subjects, who only have a solitary nodule within 3 cm in diameter, or subjects that have at most two nodules with the sum of two diameters smaller than 3 cm^21^. Cirrhosis subjects were recruited from the Dalian Sixth People’s Hospital (Dalian, China) and the First Hospital of Jilin University (Changchun, China). Detailed clinical information was presented in supplementary Tables S2 and S3.

To control the possible interference of sampling, in-house standard protocol was formulated and followed based on our previous study about the collection and storage of clinical samples^29^. All sera were stored immediately at −80 °C.

Quality control samples were used to verify the quality of metabolomics analysis. QCs were prepared by mixing same volume of each serum sample, and extracted as real samples.

### Metabolic profiling

Serum metabolic profiling was acquired using a CE-TOF/MS system (Agilent, USA) as our reported method^21^. The CE-TOF/MS analysis was operated using cation-positive (CP) mode and anion-negative (AN) mode, respectively. Instrumental details were described in supporting information.

### Data Processing and Statistics

Original data pretreatment methods were described in our previous report^21^. Briefly, procedures such as peak feature extraction, migration time correction, smoothing and alignment, were performed via the software of Qualitative analysis (B.04.00, Agilent), Quantitative Analysis (B.04.00, Agilent) and MethodMarker (Human Metabolome Technologies, Inc., HMT, Japan). To facilitate peak identification, about 500 metabolite standards were pre-analyzed by our collaborator (HMT, Japan). Then, acquired data were identified and refined based on the 80% rule to export a peak table.

Before statistical analysis, we performed the normalization for each sample using multiple internal standards, i.e., internal standard was selected independently for each metabolite to obtain the least RSD of response in all QCs. Then, data were exported to the software of SIMCA-P (Umetrics, Sweden) to develop multivariate statistical models with unit variance (UV) scaling. These visualization models include the principal component analysis (PCA) and the partial least squares discriminant analysis (PLS-DA). Response permutation test with 200 iterations was conducted to guard against the PLS-DA model overfitting. Wilcoxon Mann-Whitney test and Wilcoxon Matched-Pairs Signed-Ranks test were performed for univariate significance analysis using Multi Experiment Viewer (MeV) software (Version 4.8.1, http://www.tm4.org) and an in-house developed Matlab program (MathWorks, USA). Pathway analysis was employed on the MetaboAnalyst (http://www.metaboanalyst.ca) to identify the most relevant pathways involved in the conditions under study. To visualize the evolutionary profiles, the correlation network analysis using cytoscape software (http://www.cytoscape.org) and the hierarchical cluster analysis (HCA) using MeV software were performed. Finally, to evaluate the discriminatory capability of potential biomarkers, receiver operating characteristic curve (ROC) was exploited using the SPSS Statistics software (SPSS Inc., USA).

## Additional Information

**How to cite this article**: Zeng, J. *et al.* Metabolomics Identifies Biomarker Pattern for Early Diagnosis of Hepatocellular Carcinoma: from Diethylnitrosamine Treated Rats to Patients. *Sci. Rep.* **5**, 16101; doi: 10.1038/srep16101 (2015).

## Supplementary Material

Supporting Information

## Figures and Tables

**Table 1:** The results of ROC analysis.

		AUC	Standard Error	Sensitivity	Specificity
Animal model	(8W–14W) vs.(18W–20W)	0.918	0.044	0.929	0.857
	(8W–14W) vs.(16W–20W)	0.895	0.052	0.905	0.857
Population I	Cirrhosis vs. HCC	0.865	0.052	0.864	0.760
Population II	Cirrhosis vs.(Small HCC & General HCC)	0.905	0.034	0.760	1.000
	Cirrhosis vs. Small HCC	0.928	0.046	0.800	1.000
