# Supplementary material for: Metabolomics Identifies Biomarker Pattern for Early Diagnosis of Hepatocellular Carcinoma: from Diethylnitrosamine Treated Rats to Patients
Source: Sci Rep. 2015 Nov 3;5:16101. doi: 10.1038/srep16101 (PMC4630653; doi:10.1038/srep16101)
Supplement: Supporting Information [file srep16101-s1.doc]

**Supporting Information**

**Materials and Chemicals**

Methanol and chloroform (HPLC-grade) were purchased from Merck (Germany) and Burdick & Jackson (USA). Formic acid (98%) and ammonium acetate (> = 98%) were purchased from Sigma-Aldrich (USA). Ultrapure water (18.2 MΩ-cm, TOC = 6 ppb) was prepared from a Milli-Q system (Millipore, USA).

All chemical standards were prepared as stock solutions. 10 mM of L-methionine sulfone (Wako, Japan) and D-camphor-10-sulfonic acid sodium salt (Wako, Japan) were mixed and resolved in water (stocked Internal Standard Solution 1, ISS1). 1 mM of 3-aminopyrrolidine dihydrochloride (Aldrich, USA), N, N-diethyl-2-phenylacetamide (Wako, Japan), trimesic acid (Wako, Japan) and disodium 3-hydroxynaphthalene-2, 7-disulfonate (Wako, Japan) were mixed and resolved in methanol (stocked Internal Standard Solution 2, ISS2). Isotope standards of alanine-d3, carnitine-d3, cholic acid-d4 and succinic acid-^13^C4 were prepared as individual stock solutions.

**Serum preparation**

During the preparation, serum samples were thawed and operated on ice. Prepared methanol, containing internal standards (10 μM of stocked Internal Standard Solution 1, 0.67 μg/mL of alanine-d3, 0.5 μg/mL of carnitine-d3, 1.67 μg/mL of cholic acid-d4 and 0.8 μg/mL of succinic acid-^13^C4), were precooled for metabolites extraction. 450 μL of prepared methanol, 500 μL of chloroform and 200 μL of Milli-Q water were successively added to each sample of 50 μL serum. After each addition, the mixture was thoroughly vortexed for 1 min. Subsequently, the mixture was left to stand for 5 minutes. After centrifuging (5,000 g, 10 min at 4 °C), the upper layer was transferred. To further remove proteins, 450 μL of the upper layer was centrifugally filtered through a Millipore 5-kDa cutoff filter (USA) (13,000 g, 3h at 4°C). Finally, the filtrate from each sample was lyophilized and dissolved in Milli-Q water containing 50 μM of stocked Internal Standard Solution 2 for analysis.

Quality control (QC) samples were prepared by mixing same volume of each serum sample, and extracted as real samples.

**Instrument analysis**

Serum metabolic profiling was acquired using a CE-TOF/MS system (Agilent, USA) equipped with the coaxial sheath liquid interface. Methanol/water (50% v/v, 0.1 μM hexakis (2, 2-difluoroethoxy) phosphazene) was delivered as the sheath liquid at 10 μL/min to realize the coupling of CE and MS. This system consists of the 1260 ISO pump (G1310B), CE (G7100A), CE-electrospray ionization (ESI)-MS sprayer kit (G1607A) and TOF/MS (G6224A). The separation column was a fused silica capillary (50 μm i.d. × 80 cm) with temperature at 20 °C. The sample tray temperature was set below 5 °C using a temperature controlled minichiller (Huber, Germany).

The CE-TOF/MS analysis was operated using cation-positive (CP) mode and anion-negative (AN) mode, respectively. The detailed CE and MS methods were described in our previous reports^1,2^, and the analysis parameters were listed in supplementary Table S4.

In each analysis batch, real samples were alternated in random order, and the QCs were also inserted and analyzed in each set of 10 real samples.

**Table S1. The results of weighting**

|  | DEN group | | | | | | | | | | | | Controls | | |
| --- | --- | --- | --- | --- | --- | --- | --- | --- | --- | --- | --- | --- | --- | --- | --- |
| Week | 14 | | | 16 | | | 18 | | | 20 | | | 20 | | |
|  | Weight (g) | RW (E-02) | *p* | Weight (g) | RW (E-02) | *p* | Weight (g) | RW (E-02) | *p* | Weight (g) | RW (E-02) | *p* | Weight (g) | RW (E-02) | *p* |
| Body | 342.50 ± 45.06 |  | 0.01 | 394.43 ± 48.53 |  | 0.25 | 397.36 ± 37.98 |  | 0.42 | 419.29 ± 45.60 |  | / | 467.50 ± 35.86 |  | 0.04 |
| Liver | 18.00 ± 4.63 | 5.19 ± 1.04 | 0.64 | 20.58 ± 4.39 | 5.23 ± 1.02 | 0.42 | 24.89 ± 7.78 | 6.28 ± 1.85 | 0.42 | 23.46 ± 3.27 | 5.61 ± 0.75 | / | 14.05 ± 1.41 | 3.01 ± 0.26 | 0.00 |

RW: relative weight of liver (i.e., the normalization of liver weight to body weight); *p*: *p* values were obtained from the comparison of different groups with the DEN group (week 20) using the Wilcoxon Mann-Whitney test. The comparison of liver was based on the results of relative weight (RW).

**Table S2.** Clinical information of population I.

| Characteristics | Cirrhosis | HCC |
| --- | --- | --- |
| Number | 25 | 22 |
| Age | 53.36 ± 1.97 | 58.23 ± 2.26 |
| Sex (Male/Female) | 18/7 | 18/4 |
| AFP>20 (μg/L) |  | 10 |
| ALT (U/L) |  | 177.95 ± 49.84 |
| AST (U/L) |  | 106.90 ± 43.56 |
| HBsAg (yes/no) |  | 19/3 |
| HCV (yes/no) |  | 0/22 |
| Tumor diameter (cm)^&^ |  |  |
| <3 |  | 0 |
| 3-5 |  | 6 |
| 5-10 |  | 8 |
| >10 |  | 7 |

&: One HCC patient was lack of tumor diameter information.

ALT, alanine aminotransferase; AST, aspartate transaminase.

All data are presented as mean ± SE.

**Table S3. Clinical information of population II.**

| Characteristics | Cirrhosis | HCC | |
| --- | --- | --- | --- |
|  |  | Small HCC | General HCC |
| Number | 25 | 50 | |
|  |  | 20 | 30 |
| Age | 53.40 ± 1.46 | 50.00 ± 1.90 | 50.33 ± 1.74 |
| Sex (Male/Female) | 18/7 | 18/2 | 27/3 |
| AFP>20 (μg/L) | 8 | 15 | 14 |
| AFP (μg/L) | 52.53 ± 23.56 | 396.31 ± 104.21 | 316.18 ± 89.20 |
| GGT (U/L) |  | 57.62 ± 8.25 | 115.70 ± 22.89 |
| AKP (U/L) |  | 85.60 ± 7.43 | 109.27 ± 11.49 |
| ALT (U/L) | 95.88 ± 33.10 |  |  |
| AST (U/L) | 104.42 ± 29.98 |  |  |
| HBsAg (yes/no) |  | 18/2 | 21/9 |
| HCV (yes/no) |  | 0/20 | 1/29 |
| Primary tumor diameter (cm) |  |  |  |
| <=3 |  | 20 | 3^#^ |
| 3-5 |  |  | 11 |
| 5-10 |  |  | 9 |
| >10 |  |  | 3 |

#: These three patients were not regarded as small HCC subjects as they had more than one nodules, and the sum of tumor diameter exceeded 3cm.

γ-GT, γ-glutamyl transpeptidase; AKP, alkaline phosphatase;

All data are presented as mean ± SE.

**Table S4. The parameters of two analysis modes**

| Analysis modes | Cation-Positive (CP) | Anion-Negative (AN) |
| --- | --- | --- |
| Voltage (kV) | 27 | 30 |
| Conditioning solution | / | 25 mM ammonium acetate and 75 mM diammonium hydrogen phosphate (pH 8.5) |
| Background electrolyte | 1 M formic acid (PH 1.8) | 50 mM ammonium acetate (pH 8.5) |
| Internal pressure | / | 15mbar |
| Sample injection | 50 mbar for 3 s (about 3 nL) | 50 mbar for 25 s (about 25 nL) |
| Electrospray (ESI) mode | positive | negative |
| Nebulizer pressure (psig) | 5 | 5 |
| Dry gas temperature (°C) | 300 | 300 |
| Nitrogen flow (L/min) | 7 | 7 |
| Capillary voltage (kV) | 4 | 3.5 |
| fragmentor (V) | 105 | 125 |
| Skimmer (V) | 50 | 50 |
| Oct RFV (V) | 650 | 650 |
| Acquisition rate (spectra/s) | 1.5 | 1.5 |
| Mass range | 60-1,000 | 50-1,000 |
| Reference masses | protonated isotope of the methanol dimer ([2MeOH + H]^+^, m/z 66.0631); protonated hexakis (2, 2-difluoroethoxy) phosphazene (m/z 622.0289) | deprotonated acetic acid dimer (i.e., [2CH3COOH - H]^−^, m/z 119.0350); [hexakis (2, 2-difluoroethoxy) phosphazene + CH3COOH – H]^−^ (m/z 680.03554) |

**Table S5**. **Significant differential metabolites for the comparison between model and age-matched control rats (*p* < 0.05)**

| Mode | m/z | MT  (min) | Metabolites | Average value (Relative concentration of DEN rats to the control at the same time) | | | | | | | *p* (matched pairs sign rank) | | | | | | Class |
| --- | --- | --- | --- | --- | --- | --- | --- | --- | --- | --- | --- | --- | --- | --- | --- | --- | --- |
|  |  |  |  | 8W | 10W | 12W | 14W | 16W | 18W | 20W | 12W vs.16W | 12W vs.18W | 12W vs.20W | 14W vs.16W | 14W vs.18W | 14W vs.20W |  |
| CP | 175.1190 | 7.09 | Arg | 0.819 | 0.843 | 0.762 | 0.804 | 0.914 | 1.020 | 0.897 | 0.047 | 0.016 | 0.156 | 0.219 | 0.031 | 0.297 | Glutamate family;Urea cycle |
| CP | 291.1299 | 9.44 | Argininosuccinic acid | 0.936 | 0.946 | 1.226 | 1.475 | 1.641 | 1.789 | 1.544 | 0.078 | 0.031 | 0.078 | 0.469 | 0.156 | 0.813 | Glutamate family;Urea cycle |
| CP | 176.1030 | 11.04 | Citrulline | 1.243 | 1.260 | 1.757 | 1.828 | 1.496 | 1.631 | 1.605 | 0.109 | 0.469 | 0.469 | 0.578 | 0.938 | 0.813 | Glutamate family;Urea cycle |
| CP | 132.0768 | 8.87 | Creatine | 0.741 | 0.795 | 0.446 | 0.464 | 0.866 | 0.794 | 0.914 | 0.156 | 0.031 | 0.031 | 0.156 | 0.031 | 0.016 | Glutamate family;Urea cycle |
| CP | 114.0662 | 7.32 | Creatinine | 1.063 | 0.996 | 0.820 | 1.030 | 0.929 | 1.002 | 1.042 | 0.031 | 0.031 | 0.031 | 0.109 | 0.938 | 0.813 | Urea cycle |
| CP | 147.0764 | 10.76 | Gln | 1.209 | 1.321 | 1.021 | 1.001 | 1.096 | 1.169 | 0.994 | 0.578 | 0.078 | 0.688 | 0.469 | 0.078 | 0.813 | Glutamate family;Urea cycle |
| CP | 148.0604 | 10.94 | Glu | 0.752 | 0.909 | 0.731 | 0.984 | 0.801 | 0.681 | 1.149 | 0.219 | 0.688 | 0.078 | 0.156 | 0.016 | 0.469 | Glutamate family;Urea cycle |
| CP | 118.0611 | 8.23 | Guanidoacetic acid | 1.368 | 1.110 | 2.609 | 1.967 | 1.181 | 1.307 | 1.237 | 0.047 | 0.031 | 0.047 | 0.031 | 0.078 | 0.078 | Urea cycle |
| CP | 133.0972 | 6.84 | Ornithine | 1.386 | 1.908 | 3.420 | 3.029 | 2.310 | 2.456 | 2.620 | 0.031 | 0.016 | 0.016 | 0.109 | 0.047 | 0.219 | Glutamate family;Urea cycle |
| CP | 116.0706 | 10.82 | Pro | 0.872 | 1.019 | 1.347 | 1.252 | 1.265 | 1.483 | 1.493 | 0.297 | 0.375 | 0.813 | 0.938 | 0.219 | 0.219 | Glutamate family;Urea cycle |
| CP | 132.0655 | 12.02 | trans-4-Hydroxyproline | 1.028 | 1.072 | 1.003 | 1.150 | 1.243 | 1.389 | 1.201 | 0.078 | 0.016 | 0.109 | 0.469 | 0.078 | 0.469 | Glutamate family;Urea cycle |
| CP | 61.0396 | 20.54 | Urea-2M+H | 0.806 | 0.709 | 1.005 | 0.966 | 1.125 | 1.025 | 0.932 | 0.938 | 0.578 | 0.578 | 0.578 | 0.688 | 0.813 | Urea cycle |
| AN | 178.0510 | 8.50 | Hippuric acid | 0.542 | 0.385 | 2.193 | 0.929 | 0.711 | 1.028 | 2.160 | 0.016 | 0.016 | 0.938 | 0.016 | 0.813 | 0.031 | Urea cycle |
| AN | 167.0211 | 8.94 | Uric acid | 0.977 | 0.901 | 0.689 | 1.005 | 0.884 | 0.938 | 1.101 | 0.016 | 0.016 | 0.031 | 0.219 | 0.297 | 0.578 | Urea cycle |
| CP | 122.0270 | 11.28 | Cys | 1.186 | 0.754 | 0.706 | 0.794 | 0.788 | 0.797 | 0.841 | 0.813 | 0.469 | 0.375 | 0.938 | 0.938 | 0.578 | Serine family |
| CP | 223.0747 | 9.92 | Cystathionine | 0.755 | 0.696 | 0.991 | 1.525 | 1.615 | 1.845 | 1.604 | 0.078 | 0.047 | 0.219 | 0.813 | 0.078 | 0.938 | Serine family |
| CP | 241.0311 | 11.00 | Cystine | 1.046 | 0.862 | 0.742 | 0.838 | 0.841 | 0.852 | 0.776 | 0.297 | 0.219 | 0.469 | 0.938 | 0.938 | 0.109 | Serine family |
| CP | 120.0655 | 10.09 | Homoserine | 1.181 | 1.724 | 2.057 | 1.614 | 1.353 | 1.267 | 1.381 | 0.016 | 0.016 | 0.031 | 0.297 | 0.031 | 0.469 | Serine family derivatives |
| CP | 106.0499 | 10.01 | Ser | 0.941 | 1.026 | 0.993 | 1.049 | 1.130 | 1.204 | 1.199 | 0.016 | 0.031 | 0.078 | 0.219 | 0.078 | 0.078 | Serine family |
| CP | 90.0550 | 9.02 | Ala | 0.786 | 0.918 | 1.069 | 1.075 | 1.030 | 1.176 | 1.388 | 0.688 | 0.469 | 0.016 | 0.688 | 0.297 | 0.016 | pyruvate family |
| CP | 132.1019 | 10.33 | Leu | 0.659 | 0.708 | 0.624 | 0.766 | 0.873 | 0.922 | 0.972 | 0.031 | 0.016 | 0.016 | 0.219 | 0.078 | 0.047 | pyruvate family |
| CP | 126.0219 | 21.48 | Taurine | 0.753 | 1.024 | 0.543 | 0.832 | 0.888 | 0.823 | 0.914 | 0.016 | 0.031 | 0.078 | 0.578 | 0.813 | 0.688 | pyruvate family |
| CP | 118.0863 | 10.04 | Val | 0.678 | 0.697 | 0.720 | 0.822 | 0.884 | 0.952 | 0.974 | 0.078 | 0.016 | 0.016 | 0.578 | 0.156 | 0.109 | pyruvate family |
| CP | 133.0608 | 10.47 | Asn | 0.965 | 1.136 | 1.229 | 1.154 | 1.191 | 1.275 | 1.244 | 0.688 | 0.688 | 0.938 | 0.938 | 0.219 | 0.375 | aspartate family |
| CP | 134.0448 | 11.52 | Asp | 0.705 | 1.130 | 0.989 | 1.174 | 0.992 | 0.842 | 1.464 | 0.938 | 0.156 | 0.078 | 0.047 | 0.016 | 0.297 | aspartate family |
| CP | 132.1019 | 10.23 | Ile | 0.646 | 0.776 | 0.633 | 0.782 | 0.867 | 0.841 | 0.896 | 0.078 | 0.016 | 0.016 | 0.297 | 0.219 | 0.297 | aspartate family |
| CP | 147.1128 | 6.91 | Lys | 0.776 | 0.811 | 0.786 | 0.958 | 1.042 | 1.111 | 1.149 | 0.078 | 0.109 | 0.031 | 0.688 | 0.156 | 0.078 | aspartate family |
| CP | 150.0583 | 10.73 | Met | 1.174 | 1.024 | 1.380 | 1.165 | 0.838 | 0.914 | 1.074 | 0.031 | 0.031 | 0.219 | 0.078 | 0.156 | 0.813 | aspartate family |
| CP | 166.0532 | 11.76 | Methionine sulfoxide | 1.096 | 1.060 | 3.056 | 1.555 | 1.185 | 1.149 | 1.452 | 0.016 | 0.016 | 0.016 | 0.375 | 0.688 | 0.688 | aspartate family derivatives |
| CP | 189.1234 | 11.41 | N6-Acetyllysine | 0.870 | 0.809 | 1.067 | 1.296 | 1.346 | 1.603 | 1.414 | 0.031 | 0.016 | 0.016 | 0.938 | 0.047 | 0.297 | aspartate family derivatives |
| CP | 130.0863 | 10.30 | Pipecolic acid | 0.485 | 0.397 | 0.173 | 0.531 | 1.022 | 0.871 | 0.764 | 0.016 | 0.016 | 0.016 | 0.016 | 0.016 | 0.031 | aspartate family derivatives |
| CP | 120.0655 | 10.53 | Thr | 0.626 | 0.651 | 0.729 | 0.762 | 0.956 | 0.956 | 1.078 | 0.031 | 0.078 | 0.016 | 0.109 | 0.047 | 0.016 | aspartate family |
| CP | 156.0768 | 7.31 | His | 1.232 | 1.482 | 1.582 | 1.479 | 1.452 | 1.477 | 1.519 | 0.078 | 0.297 | 0.578 | 0.938 | 1.000 | 0.688 | Aromatic family |
| CP | 209.0921 | 9.88 | Kynurenine | 0.929 | 0.995 | 0.731 | 0.950 | 1.510 | 1.489 | 1.269 | 0.016 | 0.016 | 0.031 | 0.031 | 0.078 | 0.156 | Aromatic family derivatives |
| CP | 166.0863 | 11.10 | Phe | 1.389 | 1.619 | 1.339 | 1.118 | 1.140 | 1.069 | 1.143 | 0.219 | 0.109 | 0.219 | 1.000 | 0.938 | 0.297 | Aromatic family |
| CP | 205.0972 | 11.02 | Trp | 0.988 | 0.890 | 0.903 | 0.822 | 0.917 | 0.963 | 0.908 | 0.938 | 0.469 | 1.000 | 0.375 | 0.219 | 0.156 | Aromatic family |
| CP | 182.0812 | 11.34 | Tyr | 2.206 | 2.191 | 1.725 | 1.418 | 1.256 | 1.450 | 1.190 | 0.031 | 0.078 | 0.031 | 0.375 | 0.688 | 0.156 | Aromatic family |
| CP | 196.0968 | 9.34 | Tyrosine methyl ester | 2.239 | 2.068 | 1.807 | 1.389 | 1.207 | 1.293 | 1.108 | 0.031 | 0.031 | 0.016 | 0.109 | 0.688 | 0.078 | Aromatic family derivatives |
| CP | 139.0502 | 8.29 | Urocanic acid | 1.705 | 0.574 | 3.097 | 1.037 | 1.044 | 2.019 | 0.831 | 0.016 | 0.047 | 0.016 | 0.813 | 0.078 | 0.688 | Aromatic family |
| CP | 104.1070 | 6.88 | Choline | 0.831 | 1.060 | 1.214 | 1.151 | 0.945 | 0.950 | 1.348 | 0.031 | 0.016 | 0.375 | 0.016 | 0.078 | 0.109 | Glycerolipid metabolism |
| CP | 258.1101 | 21.10 | Glycerophosphocholine | 0.461 | 0.569 | 0.768 | 1.021 | 0.893 | 0.836 | 1.630 | 0.469 | 0.938 | 0.047 | 0.297 | 0.078 | 0.219 | Glycerolipid metabolism |
| CP | 184.0733 | 20.09 | Phosphorylcholine | 0.331 | 0.333 | 0.192 | 0.436 | 0.589 | 0.471 | 0.609 | 0.016 | 0.016 | 0.031 | 0.297 | 0.578 | 0.688 | Glycerolipid metabolism |
| AN | 140.0118 | 8.06 | Ethanolamine phosphate | 0.224 | 0.234 | 0.112 | 0.332 | 0.498 | 0.360 | 0.527 | 0.031 | 0.016 | 0.016 | 0.469 | 0.813 | 0.375 | Glycerolipid metabolism |
| AN | 105.0193 | 10.51 | Glyceric acid | 1.114 | 0.973 | 0.825 | 0.965 | 0.842 | 1.054 | 1.445 | 0.938 | 0.375 | 0.031 | 0.219 | 0.109 | 0.109 | Glycerolipid metabolism |
| CP | 118.0863 | 11.27 | Betaine | 3.201 | 3.071 | 4.548 | 2.213 | 1.320 | 1.291 | 1.606 | 0.016 | 0.016 | 0.031 | 0.016 | 0.047 | 0.375 | Choline metabolism |
| CP | 104.0706 | 10.93 | N,N-Dimethylglycine | 2.872 | 2.085 | 2.855 | 2.584 | 1.609 | 1.837 | 2.754 | 0.047 | 0.016 | 0.813 | 0.016 | 0.016 | 0.938 | Choline metabolism |
| CP | 90.0550 | 9.49 | Sarcosine | 1.458 | 0.830 | 1.757 | 1.266 | 0.920 | 3.020 | 2.699 | 0.016 | 0.813 | 0.688 | 0.016 | 0.688 | 0.047 | Choline metabolism |
| CP | 104.0706 | 9.60 | 2-Aminoisobutyric acid | 1.324 | 1.612 | 0.852 | 1.011 | 1.128 | 1.074 | 0.998 | 0.109 | 0.156 | 0.297 | 0.297 | 0.813 | 0.813 | Amino Acids and Derivatives |
| CP | 170.0924 | 7.52 | 3-Methylhistidine | 0.990 | 0.922 | 0.519 | 0.760 | 0.993 | 0.775 | 0.665 | 0.031 | 0.047 | 0.156 | 0.156 | 0.813 | 0.219 | Amino Acids and Derivatives |
| CP | 146.0924 | 8.27 | 4-Guanidinobutyric acid | 1.207 | 0.888 | 5.676 | 2.509 | 1.841 | 3.007 | 4.566 | 0.016 | 0.016 | 0.578 | 0.375 | 0.375 | 0.109 | Amino Acids and Derivatives |
| CP | 104.0706 | 7.66 | GABA | 1.558 | 2.979 | 2.880 | 2.160 | 1.728 | 1.564 | 1.116 | 0.016 | 0.031 | 0.016 | 0.469 | 0.219 | 0.031 | Amino Acids and Derivatives |
| CP | 189.1346 | 7.21 | Homoarginine | 0.902 | 0.752 | 0.746 | 0.775 | 1.189 | 1.158 | 1.020 | 0.047 | 0.078 | 0.031 | 0.047 | 0.031 | 0.031 | Amino Acids and Derivatives |
| CP | 110.0270 | 17.87 | Hypotaurine | 1.729 | 1.454 | 1.422 | 1.250 | 0.898 | 0.927 | 1.201 | 0.031 | 0.031 | 0.688 | 0.016 | 0.078 | 0.469 | Amino Acids and Derivatives |
| CP | 127.0502 | 8.03 | Imidazole-4-acetic acid | 0.813 | 0.952 | 5.906 | 3.842 | 1.795 | 3.972 | 2.868 | 0.016 | 0.109 | 0.031 | 0.078 | 0.938 | 0.688 | Amino Acids and Derivatives |
| CP | 90.0550 | 7.31 | β-Ala | 1.063 | 1.028 | 0.885 | 0.882 | 0.707 | 0.808 | 0.875 | 0.016 | 0.938 | 0.813 | 0.109 | 0.688 | 0.938 | Amino Acids and Derivatives |
| AN | 131.0462 | 9.33 | 3-Ureidopropionic acid | 28.213 | 7.756 | 6.031 | 6.178 | 10.830 | 25.119 | 5.292 | 0.047 | 0.031 | 0.688 | 0.031 | 0.031 | 0.578 | Amino Acids and Derivatives |
| AN | 174.0408 | 14.55 | N-Acetylaspartic acid | 1.261 | 0.884 | 0.692 | 0.542 | 0.725 | 0.869 | 0.815 | 0.688 | 0.156 | 0.469 | 0.156 | 0.219 | 0.297 | Amino Acids and Derivatives |
| CP | 307.0833 | 12.13 | Glutathione (GSSG)_divalent | 0.718 | 0.162 | 0.000 | 0.049 | 0.191 | 0.188 | 0.186 | 0.125 | 0.063 | 0.125 | 0.125 | 0.063 | 0.125 | Polypeptides |
| CP | 133.0608 | 8.37 | Gly-Gly | 0.803 | 1.039 | 0.724 | 1.407 | 0.879 | 0.472 | 0.530 | 0.688 | 0.063 | 0.438 | 0.219 | 0.016 | 0.031 | Polypeptides |
| CP | 189.1234 | 9.72 | Gly-Leu | 0.373 | 0.492 | 0.492 | 0.747 | 0.973 | 0.973 | 0.796 | 0.109 | 0.016 | 0.016 | 0.219 | 0.156 | 0.938 | Polypeptides |
| AN | 145.0142 | 21.31 | 2-Oxoglutaric acid | 1.377 | 1.643 | 1.001 | 1.297 | 1.085 | 1.144 | 1.405 | 0.813 | 0.688 | 0.016 | 0.047 | 0.109 | 0.469 | Central carbon metabolism |
| AN | 191.0197 | 25.97 | Citric acid | 1.248 | 1.041 | 0.898 | 1.208 | 1.208 | 1.235 | 1.123 | 0.016 | 0.031 | 0.047 | 0.938 | 0.469 | 0.813 | Central carbon metabolism |
| AN | 115.0037 | 25.02 | Fumaric acid | 1.124 | 1.122 | 0.634 | 0.835 | 0.748 | 0.893 | 1.137 | 0.219 | 0.047 | 0.016 | 0.469 | 0.813 | 0.016 | Central carbon metabolism |
| AN | 191.0197 | 28.28 | Isocitric acid | 1.208 | 1.019 | 0.843 | 1.190 | 1.221 | 1.130 | 1.143 | 0.031 | 0.016 | 0.016 | 0.469 | 0.469 | 0.938 | Central carbon metabolism |
| AN | 89.0244 | 10.76 | Lactic acid | 0.719 | 1.004 | 0.659 | 0.698 | 0.725 | 0.793 | 0.998 | 0.297 | 0.109 | 0.031 | 0.938 | 0.688 | 0.047 | Central carbon metabolism |
| AN | 87.0088 | 12.90 | Pyruvic acid | 0.831 | 1.073 | 0.504 | 0.654 | 0.709 | 0.764 | 0.957 | 0.219 | 0.031 | 0.016 | 0.688 | 0.219 | 0.031 | Central carbon metabolism |
| AN | 117.0193 | 20.95 | Succinic acid | 1.019 | 1.067 | 0.662 | 0.828 | 0.762 | 0.824 | 1.063 | 0.047 | 0.031 | 0.047 | 0.375 | 1.000 | 0.219 | Central carbon metabolism |
| CP | 180.0867 | 9.27 | Glucosamine | 0.448 | 0.679 | 1.442 | 1.156 | 0.993 | 0.573 | 1.192 | 0.938 | 0.297 | 1.000 | 0.578 | 0.047 | 1.000 | Carbohydrates |
| CP | 222.0972 | 21.54 | N-Acetylglucosamine | 1.063 | 1.413 | 0.785 | 1.399 | 1.134 | 0.750 | 1.326 | 0.297 | 0.938 | 0.047 | 0.156 | 0.047 | 0.688 | Carbohydrates |
| AN | 193.0354 | 8.14 | Glucuronic acid | 2.146 | 2.104 | 2.522 | 1.888 | 1.305 | 1.715 | 1.869 | 0.016 | 0.016 | 0.109 | 1.000 | 0.297 | 0.375 | Carbohydrates |
| AN | 103.0401 | 9.87 | 2-Hydroxybutyric acid | 1.582 | 1.690 | 0.982 | 0.970 | 0.794 | 0.859 | 0.872 | 0.219 | 0.156 | 0.813 | 0.297 | 0.469 | 0.688 | Hydroxy Acids |
| AN | 103.0401 | 9.62 | 3-Hydroxybutyric acid | 0.846 | 0.664 | 0.117 | 0.367 | 0.324 | 0.331 | 0.319 | 0.016 | 0.031 | 0.078 | 0.688 | 0.938 | 0.578 | Hydroxy Acids |
| AN | 195.0510 | 8.20 | Gluconic acid | 0.848 | 0.695 | 1.101 | 1.149 | 1.086 | 1.385 | 1.631 | 0.688 | 0.219 | 0.078 | 0.469 | 0.297 | 0.047 | Hydroxy Acids |
| AN | 209.0303 | 13.96 | Mucic acid | 0.278 | 0.327 | 0.364 | 0.373 | 0.524 | 0.581 | 0.630 | 0.156 | 0.047 | 0.047 | 0.156 | 0.016 | 0.016 | Hydroxy Acids |
| AN | 135.0299 | 9.30 | Threonic acid | 0.453 | 0.584 | 0.462 | 0.551 | 0.651 | 0.647 | 0.666 | 0.109 | 0.016 | 0.016 | 0.156 | 0.219 | 0.109 | Hydroxy Acids |
| AN | 131.0714 | 8.85 | 2-Hydroxy-4-methylvaleric acid | 1.423 | 1.362 | 2.028 | 1.474 | 1.197 | 1.433 | 1.388 | 0.047 | 0.297 | 0.156 | 0.297 | 1.000 | 0.813 | Fatty Acids |
| AN | 115.0401 | 10.21 | 2-Oxoisovaleric acid | 0.873 | 0.595 | 0.541 | 0.613 | 0.624 | 0.646 | 0.830 | 0.219 | 0.219 | 0.031 | 0.688 | 0.469 | 0.031 | Fatty Acids |
| AN | 129.0557 | 9.57 | 3/4-Methyl-2-oxovaleric acid | 0.723 | 0.487 | 0.351 | 0.397 | 0.480 | 0.461 | 0.745 | 0.031 | 0.156 | 0.016 | 0.219 | 0.469 | 0.016 | Fatty Acids |
| AN | 87.0452 | 9.94 | Butyric acid or Isobutyric acid | 1.031 | 1.032 | 5.923 | 2.085 | 1.347 | 1.948 | 2.933 | 0.016 | 0.031 | 0.109 | 0.109 | 0.688 | 0.375 | Fatty Acids |
| AN | 171.1391 | 8.01 | Decanoic acid | 0.943 | 0.789 | 0.751 | 0.966 | 0.711 | 0.811 | 0.758 | 0.578 | 0.469 | 0.813 | 0.047 | 0.109 | 0.031 | Fatty Acids |
| AN | 115.0765 | 8.99 | Hexanoic acid | 1.003 | 0.759 | 1.238 | 1.279 | 0.850 | 0.895 | 1.149 | 0.156 | 0.156 | 0.688 | 0.031 | 0.156 | 0.813 | Fatty Acids |
| AN | 157.1234 | 8.15 | Pelargonic acid | 1.046 | 0.772 | 0.876 | 1.152 | 0.766 | 1.076 | 0.731 | 0.375 | 0.156 | 0.219 | 0.016 | 0.813 | 0.016 | Fatty Acids |
| CP | 244.0928 | 9.65 | Cytidine | 0.674 | 0.821 | 0.617 | 0.754 | 0.798 | 0.707 | 0.975 | 0.219 | 0.375 | 0.031 | 0.219 | 0.375 | 0.109 | Nucleosides |
| CP | 112.0505 | 7.26 | Cytosine | 0.765 | 0.439 | 2.656 | 1.634 | 4.235 | 4.138 | 4.326 | 0.563 | 0.313 | 0.219 | 0.219 | 0.078 | 0.078 | Nucleosides |
| CP | 245.0768 | 21.53 | Uridine | 0.736 | 0.684 | 0.789 | 0.978 | 1.141 | 1.045 | 0.911 | 0.031 | 0.047 | 0.375 | 0.078 | 0.578 | 0.813 | Nucleosides |
| CP | 159.0513 | 21.47 | Allantoin | 0.828 | 0.638 | 0.876 | 1.127 | 1.143 | 1.354 | 1.209 | 0.047 | 0.031 | 0.016 | 0.938 | 0.375 | 0.578 | Amines |
| CP | 162.1125 | 8.53 | Carnitine | 0.685 | 0.629 | 0.583 | 0.599 | 0.653 | 0.670 | 0.854 | 0.469 | 0.469 | 0.047 | 0.375 | 0.375 | 0.047 | Amines |
| CP | 204.1230 | 9.03 | O-Acetylcarnitine | 0.647 | 0.590 | 0.230 | 0.377 | 0.475 | 0.491 | 0.467 | 0.031 | 0.016 | 0.016 | 0.031 | 0.109 | 0.297 | Amines |
| AN | 218.1034 | 7.64 | Pantothenic acid | 1.240 | 1.429 | 1.306 | 1.467 | 1.246 | 1.590 | 1.917 | 1.000 | 0.156 | 0.031 | 0.031 | 0.578 | 0.016 | Amines |
| AN | 407.2803 | 6.89 | Cholic acid | 7.518 | 8.355 | 2.414 | 6.661 | 3.243 | 4.587 | 2.381 | 0.938 | 0.938 | 0.688 | 0.156 | 0.688 | 0.047 | Bile acids |
| AN | 464.3018 | 6.77 | Glycocholic acid | 42.078 | 28.154 | 7.902 | 25.183 | 10.120 | 19.075 | 4.544 | 0.578 | 0.578 | 0.375 | 0.016 | 0.578 | 0.016 | Bile acids |
| CP | 228.0979 | 9.42 | 2'-Deoxycytidine | 0.780 | 0.930 | 1.037 | 1.008 | 1.027 | 1.106 | 1.022 | 0.688 | 0.109 | 0.938 | 0.938 | 0.219 | 0.813 | Purines and Pyridines |
| CP | 242.1135 | 9.75 | 5-Methyl-2'-deoxycytidine | 1.402 | 1.230 | 2.073 | 1.511 | 1.196 | 1.378 | 1.177 | 0.031 | 0.031 | 0.047 | 0.297 | 0.813 | 0.688 | Purines and Pyridines |
| CP | 123.0553 | 7.40 | Nicotinamide | 0.730 | 0.792 | 0.531 | 0.814 | 0.671 | 0.710 | 0.908 | 0.219 | 0.156 | 0.047 | 0.469 | 0.578 | 0.578 | Purines and Pyridines |
| CP | 137.0709 | 7.35 | 1-Methylnicotinamide | 3.568 | 1.813 | 1.452 | 3.073 | 1.564 | 1.986 | 2.103 | 0.578 | 0.219 | 0.297 | 0.047 | 0.109 | 0.375 | others |
| CP | 175.1077 | 11.45 | N5-Ethylglutamine | 0.624 | 0.505 | 0.630 | 0.837 | 0.943 | 0.836 | 0.721 | 0.078 | 0.156 | 0.688 | 0.156 | 0.688 | 0.156 | others |
| CP | 177.1022 | 8.78 | Serotonin | 0.830 | 0.931 | 0.582 | 0.924 | 1.029 | 0.990 | 0.816 | 0.016 | 0.031 | 0.297 | 0.469 | 0.578 | 0.219 | others |
| CP | 148.0604 | 12.28 | threo-B-Methylaspartic acid | 0.688 | 0.493 | 0.314 | 0.307 | 0.303 | 0.487 | 0.438 | 0.938 | 0.219 | 0.109 | 0.813 | 0.109 | 0.047 | others |
| CP | 138.0550 | 10.46 | Trigonelline | 0.589 | 0.484 | 2.982 | 1.382 | 1.600 | 1.554 | 1.879 | 0.016 | 0.016 | 0.016 | 0.469 | 0.297 | 0.156 | others |
| CP | 76.0757 | 6.61 | Trimethylamine N-oxide | 0.475 | 0.454 | 3.508 | 1.205 | 0.875 | 1.145 | 2.961 | 0.016 | 0.016 | 0.375 | 0.219 | 0.938 | 0.047 | others |
| CP | 191.1026 | 9.52 | Unknown 1 | 1.655 | 3.268 | 5.217 | 3.719 | 2.417 | 2.306 | 2.423 | 0.016 | 0.016 | 0.016 | 0.016 | 0.016 | 0.031 | others |
| CP | 177.0982 | 8.80 | Unknown 10 | 0.824 | 0.924 | 0.592 | 0.923 | 1.026 | 0.986 | 0.819 | 0.016 | 0.031 | 0.297 | 0.375 | 0.813 | 0.297 | others |
| CP | 136.0427 | 11.55 | Unknown 3 | 0.822 | 1.128 | 1.211 | 1.689 | 1.421 | 1.585 | 1.899 | 0.469 | 0.578 | 0.156 | 0.031 | 1.000 | 0.375 | others |
| CP | 146.1176 | 8.12 | Unknown 5 | 1.820 | 1.874 | 1.733 | 1.313 | 1.053 | 1.013 | 1.102 | 0.016 | 0.016 | 0.031 | 0.156 | 0.156 | 0.297 | others |
| CP | 189.1346 | 6.01 | Unknown 6 | 0.902 | 0.817 | 0.824 | 0.746 | 0.955 | 0.961 | 0.804 | 0.078 | 0.047 | 0.813 | 0.156 | 0.031 | 0.578 | others |
| CP | 176.0666 | 10.34 | Unknown 9 | 0.575 | 0.672 | 0.662 | 0.736 | 0.951 | 0.760 | 0.869 | 0.016 | 0.219 | 0.219 | 0.297 | 1.000 | 0.469 | others |
| AN | 101.0244 | 10.98 | Acetoacetic acid | 1.281 | 0.732 | 0.502 | 0.624 | 0.360 | 0.731 | 0.500 | 0.469 | 0.297 | 0.813 | 0.109 | 0.578 | 0.469 | others |
| AN | 121.0295 | 9.94 | Benzoic acid | 1.755 | 1.175 | 2.150 | 1.167 | 1.242 | 1.527 | 0.808 | 0.016 | 0.031 | 0.016 | 1.000 | 0.078 | 0.297 | others |
| AN | 130.0510 | 9.02 | N-Acetyl-β-alanine | 0.898 | 0.991 | 1.052 | 1.225 | 1.134 | 1.132 | 1.433 | 0.156 | 0.219 | 0.016 | 0.375 | 0.813 | 0.156 | others |
| AN | 73.0295 | 11.15 | Unknown 2 | 1.205 | 1.755 | 2.360 | 1.900 | 1.226 | 1.294 | 1.493 | 0.078 | 0.031 | 0.109 | 0.078 | 0.219 | 0.688 | others |
| AN | 182.0459 | 9.48 | Unknown 4 | 0.851 | 0.901 | 0.684 | 0.966 | 0.960 | 0.756 | 0.723 | 0.219 | 0.813 | 0.688 | 1.000 | 0.156 | 0.078 | others |
| AN | 147.0452 | 8.79 | Unknown 7 | 0.754 | 0.813 | 0.744 | 1.001 | 0.844 | 0.587 | 1.219 | 0.156 | 0.156 | 0.078 | 0.297 | 0.016 | 0.469 | others |
| AN | 190.0543 | 7.95 | Unknown 8 | 1.470 | 1.790 | 1.465 | 1.102 | 1.152 | 0.967 | 1.144 | 0.219 | 0.031 | 0.219 | 1.000 | 0.109 | 0.813 | others |


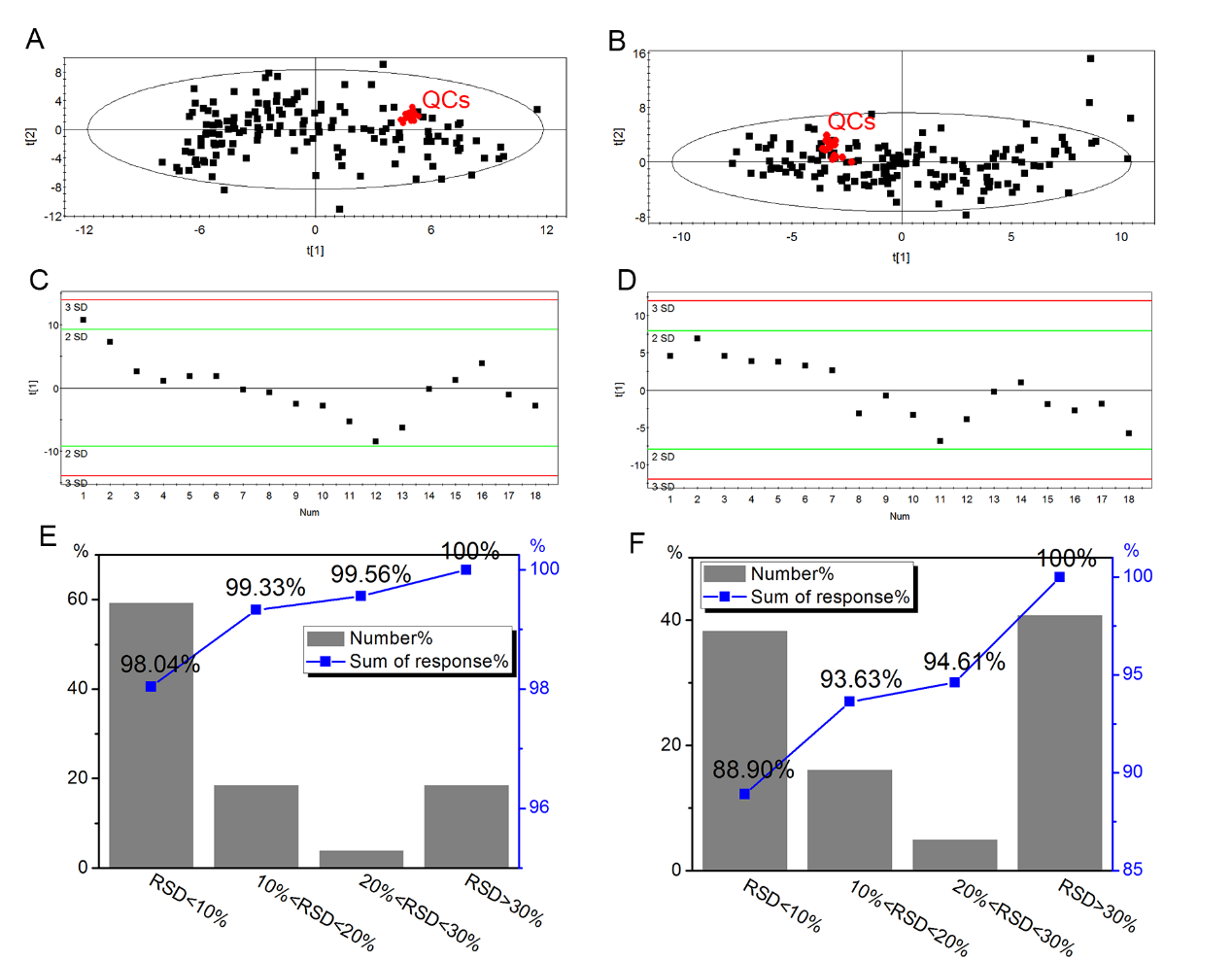


**Figure S1. Evaluation of analytical characteristics.** (A) and (B) are PCA score plots for all samples in CP and AN mode, respectively. (C) and (D) are PCA score plots for QC samples in CP and AN mode, respectively. (E) and (F) present the distributions of RSD for metabolites among all QC samples in CP and AN mode, respectively. Each column represents the percentage of compound number within corresponding RSD range. The line reveals the accumulation of percentage of responses.


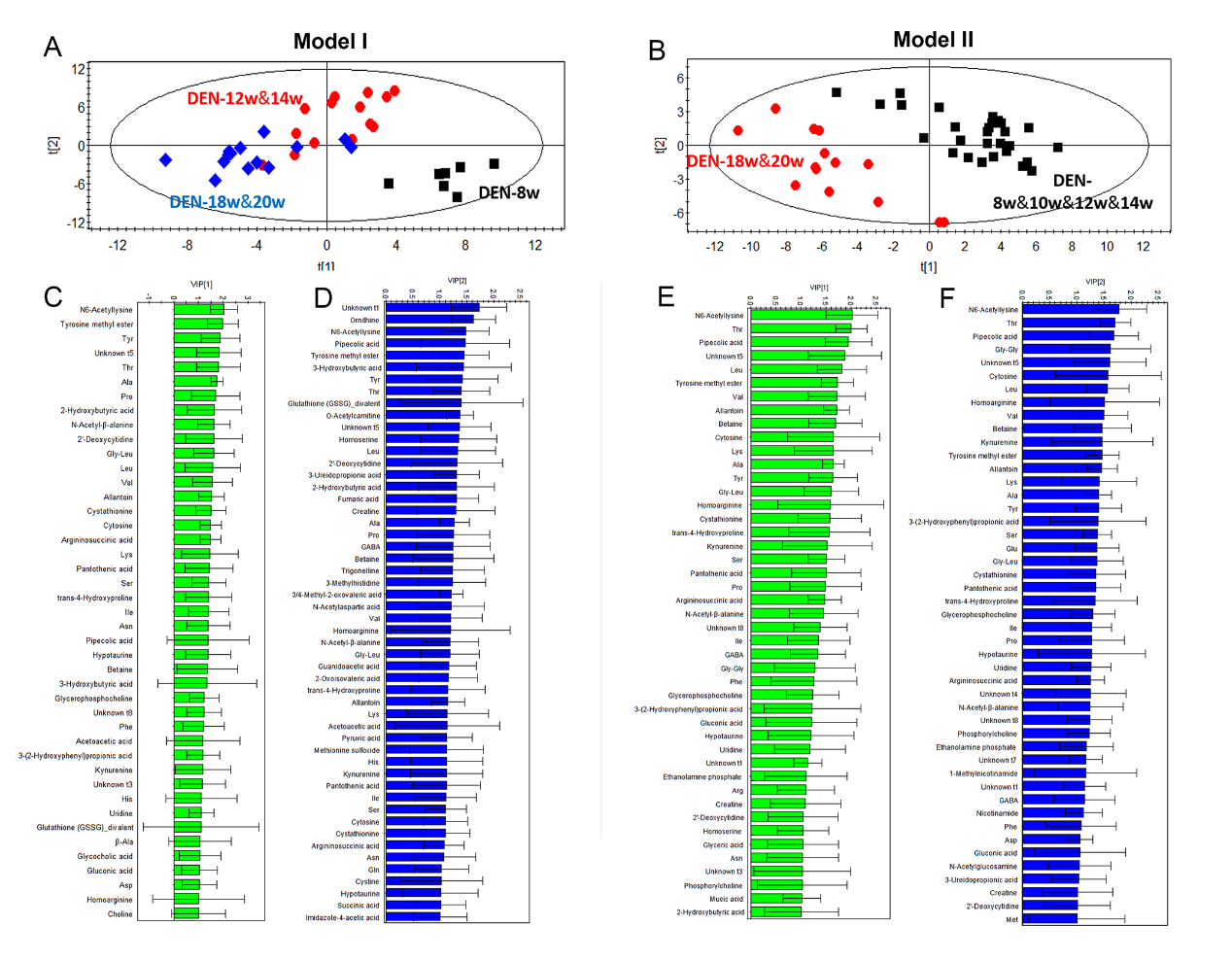


**Figure S2. Multivariate statistical analysis.** Two PLS-DA models were developed using the conversion dataset (i.e., the contents of metabolites for each DEN sample divided by the average of age-matched controls) with all variables UV scaled. **For model I,** (A) are score plot for the classification of inflammation (week 8), cirrhosis (weeks 12 to 14) and HCC (weeks 18 to 20) samples. Samples from interfacial stages (week 10 and week 16) were not included. 43 and 52 metabolites with VIP > 1 were presented in the column plots of VIP value with jack-knifed confidence intervals for the first and second principal component, respectively. A total of 63 metabolites were refined from model I (i.e., VIP value exceeding 1 for either principal components). **For model II,** (B) are score plot for the categorization of pre-HCC (weeks 8-14) and HCC (weeks 18-20) samples. Samples from week 16 were not included. 45 and 47 metabolites with VIP > 1 were listed in the column plots of VIP value with jack-knifed confidence intervals for the first and second principal component, respectively. A total of 54 metabolites were refined from model II. Based on response permutation test with 200 iterations, these PLS-DA model were validated without overfitting.

**REFERENCES**

1 Zeng, J. *et al.* Effect of bisphenol A on rat metabolic profiling studied by using capillary electrophoresis time-of-flight mass spectrometry. *Environ. Sci. Technol.* **47**, 7457-7465, doi:10.1021/es400490f (2013).

2 Zeng, J. *et al.* Metabolomics Study of Hepatocellular Carcinoma: Discovery and Validation of Serum Potential Biomarkers by Using Capillary Electrophoresis–Mass Spectrometry. *J. Proteome Res.*, doi:10.1021/pr500390y (2014).
